# Fabrication of hollow PbS nanospheres and application in phenol release

**DOI:** 10.1186/2193-1801-2-323

**Published:** 2013-07-18

**Authors:** Jian Ye, Lanping Sun, Shengping Gao

**Affiliations:** Department of Chemical and Environmental Engineering, Bengbu College, Bengbu, 233030 China

**Keywords:** Hollow PbS nanospheres, Lead dimethacrylate, ATRP, Release

## Abstract

This article demonstrates a versatile method to prepare the hollow PbS nanospheres via the template method. First, the latex poly (vinyl benzyl chloride) (PVBC) nanoparticles were synthesized by the radical polymerization, followed by the atom transfer reversible polymerization of lead (II) dimethacrylate (Pb (MA)_2_) on the surfaces of the latex nanoparticles. Then, the ethanethioamide was reacted with the nanoparticles to afford the PbS. By calcination at 600°C for 6 h, the template was removed to obtain the hollow PbS nanospheres. The structure, morphology and optical properties of the hollow PbS nanospheres were carefully investigated. The received hollow PbS nanospheres could be used for the controlled release of phenol after absorbing phenol solution.

## Introduction

Semiconductor materials have been the focus in material science, due to their prominent optical and electronic properties and their potential applications for the preparation of optical signal processors and switches (Ridley et al. 1999). As an important IV–VI group semiconductor, lead sulfide (PbS) has attracted considerable attention for many decades due to its specially small band gap (0.41 eV at 300 K) and a larger exciton Bohr radius of 18 nm (Machol et al. 1993). What is more, a blue shift from near infrared to visible region can occur. This kind of material presented fantastic optical and electric properties, which could be potentially applied widely in optoelectronic materials such as inductor, infrared detector, photoelectric converter and solar cells (McDonald et al. 2005; Deng et al. 2009; Gao et al. 2011; Lee et al. 2008; Noone et al. 2010; Asunskis et al. 2008; Jain et al. 2010). The design and fabrication of PbS nanostructure have attracted considerable attention in recent years. The synthesis of closed nanorods (Wang & Yang 2000), nanowires (Lau et al. 2009) and dendritic structures (Zhou et al. 2006) by different methods such as solvothermal, microwave irradiation and thermal decomposition have been reported by many groups. Additionally, PbS crystals with other morphologies have also been reported (Lee et al. 2002; Peng et al. 2008).

Recently, hollow micro- or nanospheres have attracted much attention because of their specific structures and potential applications. Owing to their low density, large surface area, and surface permeability, hollow spheres were widely used as artificial cell, catalysts, fillers, and capsules for controlled release of drugs and dyes (Huang et al. 1999; Caruso 2000). The hollow nanospheres could be often prepared via the template methods, such as the one-pot preparation of hollow silica spheres by using thermosensitive poly (N-isopropylacrylamide) as a reversible template (Du et al. 2009), and the fabrication of polymer nanocapsules with cross-linked organic–inorganic hybrid walls (Chen et al. 2012). The hollow PbS nanospheres have been obtained via the sonochemical synthesis method (Wang et al. 2006) and the block copolymer micro-emulsion based approach (Ding et al. 2007; Gao et al. 2005). However, few articles reported on the synthesis of hollow PbS nanospheres prepared with the assistance of any surfactant and atom transfer reversible polymerization (ATRP) process. Furthermore, the application of hollow PbS nanospheres in the field of controlled release needs to be explored.

In this paper, a simple chemical precipitation route was demonstrated for the fabrication of poly (vinyl benzyl chloride)@PbS (PVBC@PbS) core-shell and hollow PbS nanospheres. The structure, morphology and optical properties of the hollow nanospheres were investigated. The hollow PbS nanospheres embedded with phenol could be used for the controlled release of phenol. It is worth noting that the method demonstrated in this article may potentially find useful application in the field of controlled release.

## Experimental work

### Materials

The vinyl benzyl chloride (VBC), polyethylene-polypropylene glycol, sodium dodecylbenzenesulphonate, azobisisobutyronitrile (AIBN) and ethanethioamide were purchased from Sinopharm Chemical Reagent Corporation Ltd. The *p*-toluenesulfonylchloride and 2, 2'-dipyridyl (Bpy, >99%) were purchased from ACROS. The solvents were purchased from Tianjin Fuchen Chemical Reagent Factory. The chemicals were used as received without further purification. Copper (I) chloride (CuCl) was purified according to procedures described in the literature (Matyjaszewski et al. 1999). Lead dimethacrylate (LDMA) was synthesized according to the literature (Dave 1984) and re-crystallized from ethanol.

### Preparation of poly (vinyl benzyl chloride) (PVBC) latex nanospheres

Vinyl benzyl chloride (21.4 g, 0.14 mol), polyethylene-polypropylene glycol (4.0 g), sodium dodecylbenzenesulphonate (0.5 g, 1.43 mmol) and deionized water (74.0 mL) were charged into a 250 mL flask. After vigorous stirring for 30 min, the AIBN (0.065 g, 0.39 mmol) was added and the reaction mixture was heated to 65°C and stirred for 10 h. Then, the mixture was poured into a 500 mL beaker containing ethanol. The precipitated polymer was filtered and extracted with ethanol and deionized H_2_O for 6 h. The PVBC nanospheres were dried in vacuum at 80°C for 12 h and isolated as white solid in a weight yield of 87%, and the result could be reproduced by the careful control of the reaction conditions.

### Surface-initiated atom transfer radical polymerization

Into a Pyrex tube, the PVBC latex nanospheres (0.04 g) were dispersed in 40 mL of ethanol solution containing LDMA (3.2 g, 8.5 mmol) and *p*-toluenesulfonylchloride (64.8 mg, 0.34 mmol). The mixture was purged by nitrogen for 30 min, followed by the addition of CuCl (33.7 mg, 0.34 mmol) and Bpy (106.2 mg, 0.34 mmol). The CuCl and Bpy were used as the catalyst in the atom transfer radical polymerization. The Pyrex tube was sealed and kept at 90°C for several hours after purging with argon for another 10 min. The poly (vinyl benzyl chloride) grafted poly (lead dimethacrylate) (PVBC-g-PLDMA) nanospheres were collected by centrifuging at 20000 rpm. The collected nanospheres were re-dispersed in ethanol and centrifuged at 1000 rpm for 5 min to remove any Cu (II) precipitate formed in the ATRP process. The nanospheres were then collected by centrifuging at 20000 rpm and subjected to repeated cycles of washing with ethanol and centrifugation to remove the untreated monomer and homo-polymer before further characterization.

### Reaction with ethanethioamide

The PVBC-g-PLDMA nanospheres were dispersed into 10 mL ethanol in a 100 mL flask. Ethanethioamide (2.25 g, 0.03 mol, 3 M solution in ethanol) was added in 3 h under vigorous stirring. The reaction mixture was stirred for additional 12 h, and the precipitation was isolated with centrifugation and washed with ethanol for 5 times. The received PVBC@PbS nanospheres were dried in vacuum at 40°C for 24 h.

### Removing the PVBC templates by calcination

The PVBC@PbS core-shell hybrid nanospheres were added into quartz dish and then put into tubular-furnace, after vacuum pumping and circulated by argon. The temperature was raised to 600°C and stood for 6 h, followed by cooling to room temperature. The nanospheres were dispersed into 50 mL THF in a 100 mL flask and stirred for 12 h. The mixture was filtered and washed with deionized water for 3 times. The received hollow PbS nanospheres were dried in vacuum at 40°C for 24 h.

### Release behavior of hollow PbS nanospheres loaded with phenol

Due to the sensitive channel pathway in hollow PbS nanospheres, the controlled release behavior of hollow PbS nanospheres loaded with phenol was investigated. The hollow PbS nanospheres embedded with phenol was prepared by three different ways. First, 100 mg of hollow PbS nanospheres was added into 20 mL phenol aqueous solution (0.01 M). The hollow PbS nanospheres were then collected by centrifugation after incubation at room temperature for 24 h and washing by deionized water for 5 times. Second, hollow PbS nanospheres (100 mg) were heated to 300°C under nitrogen and then added into 20 mL phenol aqueous solution (0.01 M) directly. The nanospheres were then collected after stirring for 30 min and washed by deionized water for 5 times. Third, hollow PbS nanospheres (100 mg) were added into 20 mL phenol aqueous solution (0.01 M) and stirred for 3 h under vacuum at room temperature. The nanospheres were collected after washing by deionized water for 5 times. The absorbance of the solution was measured by the UV spectrophotometer, and the phenol concentration was calculated by the use of the standard curves.

The hollow PbS nanospheres embedded with phenol were added into dialysis bags, followed by the soaking in 50 mL deionized water under stirring. The solution was measured by the UV spectrophotometer at wavelength of 268 nm. After 36 h, the data were collected and analyzed in comparison with the release behavior of pristine phenol.

### Characterization

The fourier transform infrared resonance (FTIR) spectra of the samples dispersed in KBr disks were recorded on a SHIMADZU IRprestige-21 spectrophotometer. Transmission electron microscope (TEM) analysis was used to characterize the morphology of the nanospheres. In a typical experiment, several drops of the colloidal dispersion were introduced onto a carbon film supported by a copper grid. The droplet was allowed to dry in air, and then observed under a JEM-2010 (HR) transmission electron microscope operating at an acceleration voltage of 100 kV. The X-ray diffraction (XRD) study of the samples was carried out on a Bruker D8 Focus X-ray diffractometer, operating at 40 kV and 40 mA with a copper target (λ = 1.54 Å) and at a scanning rate of 2°/min. The ultraviolet–visible spectra of the samples were obtained with a Hitachi UV-2300 spectrophotometer. The photoluminescence (PL) of the samples was measured on a Shimadzu RF-5301PC spectrofluorophotometer. Both experiments were performed at the ambient temperature.

## Results and discussion

**Scheme 1:**
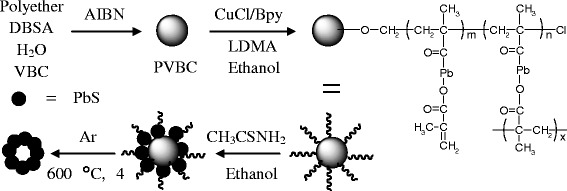
**The synthetic process for the preparation of hollow PbS nanospheres with sensitive channel pathway in shell.**

The synthetic process of hollow PbS nanospheres was illustrated in Scheme 1. The PVBC latex nanoparticles were obtained by the radical polymerization of VBC initiated by the AIBN in the presence of surfactants. Then, the chloro atoms on the surfaces of PVBC nanoparticles initiated the ATRP of LDMA to afford the PVBC-g-PLDMA. The obtained PVBC-g-PLDMA nanospheres were then reacted with ethanethioamide, leading to the formation of PVBC@PbS core-shell nanospheres. After removal of the PVBC templates by calcination, the hollow PbS nanospheres were then received. The corresponding release behavior of phenol adsorbed by hollow PbS nanospheres was investigated. It is noted that the distribution of benzyl chloride groups on the surfaces of PVBC latex nanoparticles was not homogeneous, which could lead to the formation of PLDMA with different densities. After the reaction, the PbS nanoparticles with different diameters and densities were distributing on the surfaces of the PVBC latex nanoparticles, leading to the formation of PbS shell by removal of the inner PVBC matrix.

### Structure analysis

**Figure 1:**
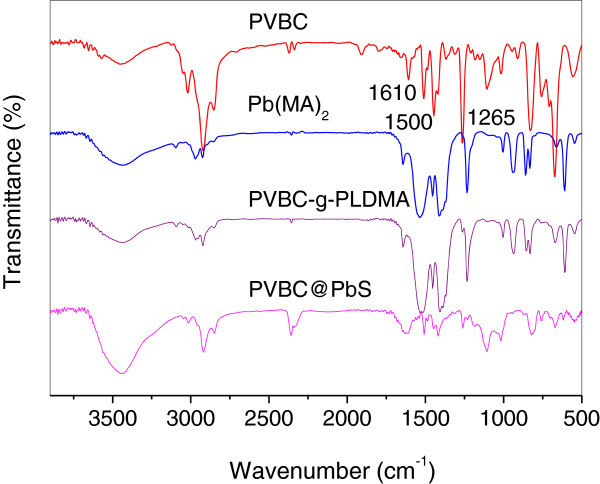
**FTIR spectra of PVBC, Pb(MA)**_**2**_**, PVBC-g-PLDMA and PVBC@PbS hybrid nanospheres.**

Figure 1 shows the IR spectra of the specimens. For the pristine PVBC, the peaks at 1610 and 1500 cm^-1^ were ascribed to the absorption of benzene ring. The peaks at 3100, 2950, 2860 and 1380 cm^-1^ were attributed to C-H stretching vibrations of benzene ring, aliphatic segments, and C-H shear vibration, respectively. Furthermore, the absorption peak at 1265 cm^-1^ corresponded to the –CH_2_Cl group. It is indicated that the PVBC has been successfully obtained during the reaction process. For the Pb(MA)_2_ monomer, the peaks at 1515 and 1450 cm^-1^ corresponded to the asymmetric carboxylate vibration, and the peak at 1387 cm^-1^ was ascribed to the symmetric carboxylate vibration. After the grafting of PLDMA onto PVBC, the characteristic absorption peaks attributed to both of PVBC and Pb(MA)_2_ were observed. By the treatment of ethanethioamide, the relative intensity of absorption peaks at 1520 cm^-1^ significantly decreased, due to the formation of PbS during the reaction. Thus, the amount of carboxylate groups decreased, leading to the decrease of the relative intensity of the absorption peaks at 1520 cm^-1^. The PVBC@PbS nanospheres were successfully obtained after the reaction process.

**Figure 2:**
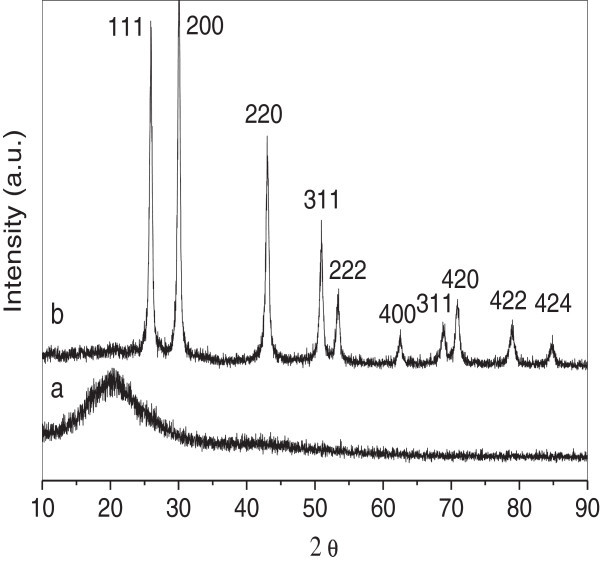
**XRD spectra of (a) PVBC and (b) hollow PbS nanospheres, respectively.**

The X-ray spectrum was further performed to investigate the crystalline structure of PVBC and hollow PbS nanospheres. As shown in Figure 2, the PVBC nanospheres exhibited a broad reflection peak at 2θ = 20.0°, indicating that PVBC was amorphous. In contrast, the hollow PbS nanospheres showed sharp reflection peaks, indicating that the nanospheres were highly crystalline. The strong diffraction peaks corresponded to the face-centered cubic of PbS (111), (200), (220), (311), (222), (400), (331) crystal face consistent with powder diffraction card (PDF No.5-0592). The above results indicate that the PbS nanospheres have been successfully prepared. Based on the Scherrer equation, the diameter of the hollow PbS nanospheres was calculated to be about 15.0 nm.

### Morphology

**Figure 3:**
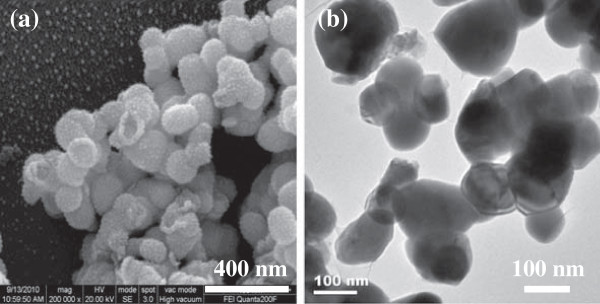
**(a) SEM and (b) TEM graphs of hollow PbS nanospheres, respectively.**

Both of SEM and TEM analysis were used to observe the morphology of hollow PbS nanospheres. As shown in Figure 3a, the broken PbS nanospheres could be observed. The diameter of the nanospheres ranged from 80 to 110 nm, and the average size was determined to be about 100 nm. The thickness of the shell was about 20 nm. Furthermore, in Figure 3b, the color depth of grain of the nanospheres was inconsistent, indicating of the hollow structure in nanospheres. In addition, some of the nanospheres exhibited peanut-shaped hollow structure, which might be due to the adjacent nanospheres collided together during the reaction process. It is concluded that the hollow PbS nanospheres were obtained.

### Optical properties

**Figure 4:**
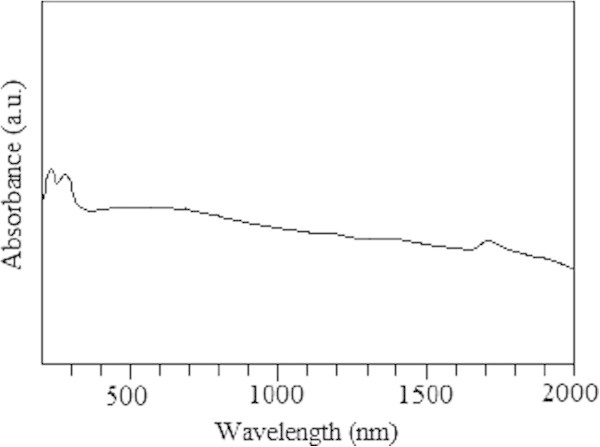
**UV–vis-NIR spectra of hollow PbS nanospheres dispersing in ethanol.**

**Figure 5:**
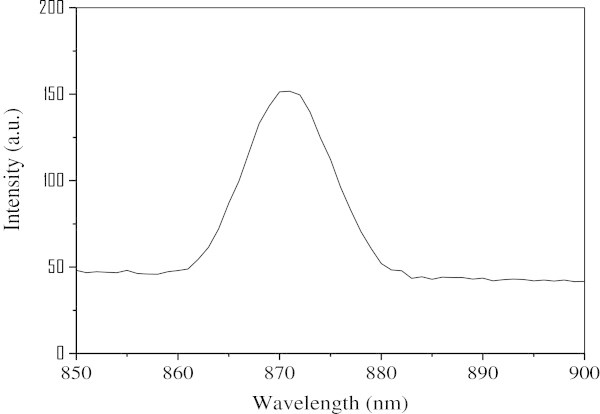
**Photoluminescence spectrum of the hollow PbS nanospheres in ethanol excited at 872 nm.**

Figure 4 shows the typical reflectance UV–vis spectra for hollow PbS nanospheres. The absorption edge started from the near infrared region, and the absorption region was about 1800 nm (band gap 0.69 eV), showing of an obvious blue shift in contrast to absorption region at 3200 nm for PbS (band gap 0.42 eV). It is indicated that the significant quantum size effect could be noticed. In the photoluminescence spectra (Figure 5), the hollow PbS nanospheres emitted at 872 nm when excited at 580 nm. Nano-sized semiconductor particles generally exhibited a threshold energy in the optical absorption measurements, due to the size-specific band gap structures, which could be affected by the blue shifting of the absorption edge with decreasing particle size. This provided an indirect way to evaluate, at least quantitatively, the variation of particle core dimensions. Therefore, it is concluded that the hollow PbS nanospheres exhibited a rather small size, which was consistent with the results in SEM and TEM observation. The UV–vis absorption edge showed a very significant blue shift from the bulk PbS crystals, indicating the quantum confinement.

### Release behavior of phenol adsorbed by hollow PbS nanospheres

The standard curve between concentration and absorbance (determined by UV spectra) for the phenol aqueous solution has been carefully prepared. It is revealed that a good linear relationship could be obtained at the phenol concentration below 30 mg/L. Based on the standard curve, the phenol concentration in solution could then be calculated after the measurement of absorbance in solution.

**Figure 6:**
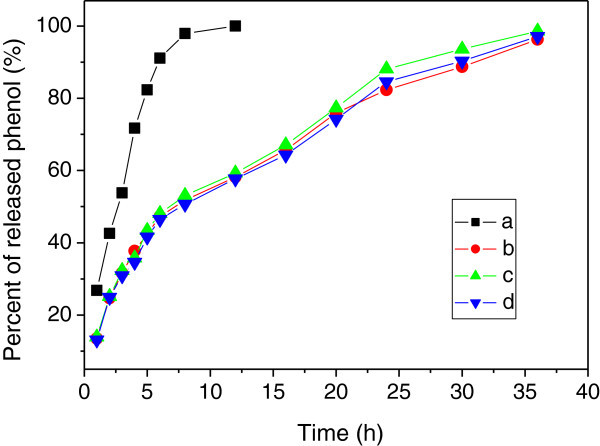
**The curves of the percent of phenol released from the dialysis bags versus time, (a) pristine phenol, and hollow PbS nanospheres loaded phenol prepared at room temperature (b), prepared under vacuum (c), prepared after heating the nanospheres to 300°C (d), respectively.**

The quality of phenol adsorbed by the hollow PbS nanospheres was dependent on the preparation conditions. The percent of loaded phenol encapsulated by hollow PbS nanospheres was 61.9%, 60.8% and 76.3% for the specimens prepared at room temperature, quenching and vacuum conditions, respectively. The sample after treating by the vacuum absorbed more content of phenol than the other methods. It is suggested that the phenol could penetrate into the hollow nanospheres via the shell. Figure 6 shows the release behavior of phenol adsorbed by hollow PbS nanospheres which were prepared at different conditions. For the pristine phenol, about 35% phenol released from the dialysis bag after 3 h, and 80% phenol released after 5 h. In comparison, the hollow PbS nanospheres released the phenol in a much lower rate. About 50% of the phenol released from the dialysis bag after 8 h. It is noticed that the phenol release rate was almost constant and exhibited a controlled releasing behavior. Almost 95% phenol released after 36 h, and the releasing time was much longer than that of pristine phenol. Therefore, the hollow PbS nanospheres could potentially be used for the controlled release of drugs.

## Conclusions

The hollow PbS nanospheres have been successfully fabricated via the convenient template method, including the preparation of PVBC latex nanoparticles by radical polymerization, grafting of PLDMA onto PVBC via ATRP, and removal of the polymer template by calcination. The hollow PbS nanospheres were highly crystalline and exhibited the quantum confinement due to the smaller average size of PbS nanospheres than that of the excitonic Bohr radius of the bulk PbS. The hollow PbS nanospheres could be used for the controlled release of phenol, which might be contributing to the existence of PbS nanosphere shell.

## References

[CR1] Asunskis DJ, Bolotin IL, Hanley L (2008). Nonlinear optical properties of PbS nanocrystals grown in polymer solutions. J Phys Chem C.

[CR2] Caruso DF (2000). Hollow capsule processing through colloidal templating and self-assembly. Chem A Eur J.

[CR3] Chen TY, Du BY, Fan ZQ (2012). Facile fabrication of polymer nanocapsules with cross-linked organic–inorganic hybrid walls. Langmuir.

[CR4] Dave AM (1984). Synthesis of lead dimethacrylate. Polymer.

[CR5] Deng DW, Zhang WH, Chen XY, Liu F, Zhang J, Gu YQ, Hong JM (2009). Facile synthesis of high-quality, water-soluble, near-infrared-emitting PbS quantum dots. Eur Polym J Inorg Chem.

[CR6] Ding YH, Liu XX, Guo R (2007). Synthesis of hollow PbS nanospheres in plutonic F127/cyclohexane/H_2_O microemulsions. Colloids Surf A Physicochem Eng Aspects.

[CR7] Du BY, Cao Z, Li ZB, Mei AX, Zhang XH, Nie JJ, Xu JT, Fan ZQ (2009). One-pot preparation of hollow silica spheres by using thermosensitive poly (N-isopropylacrylamide) as a reversible template. Langmuir.

[CR8] Gao JB, Luther JM, Semonin OE, Ellingson RJ, Nozik AJ, Beard MC, Wang Y, Xia CY (2005). Monodisperse spherical colloids of Pb and their use as chemical templates to produce hollow particles. Adv Mater.

[CR9] Gao JB, Luther JM, Semonin OE, Ellingson RJ, Nozik AJ, Beard MC (2011). Quantum dot size dependent J-V characteristics in heterojunction ZnO/PbS quantum dot solar cells. Nano Lett.

[CR10] Huang HY, Remsen EE, Kowalewski T, Wooley KL (1999). Nanocages derived from shell cross-linked micelle templates. J Am Chem Soc.

[CR11] Jain PK, Amirav L, Aloni S, Alivisatos AP (2010). Nanoheterostructure cation exchange: Anionic framework conservation. J Am Chem Soc.

[CR12] Lau YKA, Chernak DJ, Bierman MJ, Jin S (2009). Formation of PbS nanowire pine trees driven by screw dislocations. J Am Chem Soc.

[CR13] Lee SM, Jun YW, Cho SN, Cheon JW (2002). Single-crystalline star-shaped nanocrystals and their evolution: Programming the geometry of nano-building blocks. J Am Chem Soc.

[CR14] Lee JS, Shevchenko EV, Talapin DV (2008). Au-PbS core-shell nanocrystals: Plasmonic absorption enhancement and electrical doping via intra-particle charge transfer. J Am Chem Soc.

[CR15] Machol JL, Wise FW, Patel RC (1993). Vibronic quantum beats in PbS microcrystallites. Phys Rev B.

[CR16] Matyjaszewski K, Miller PJ, Shukla N, Immaraporn B, Gelman A, Luokala BB, Siclovan TM, Kickelbick G, Vallant T, Hoffmann H, Pakula T (1999). Polymers at Interfaces: Using atom transfer radical polymerization in the controlled growth of homopolymers and block copolymers from silicon surfaces in the absence of untethered sacrificial initiator. Macromolecules.

[CR17] McDonald SA, Konstantatos G, Zhang S, Cyr PW, Klem EJD, Levina L, Sargent EH (2005). Solution-processed PbS quantum dot infrared photodetectors and photovoltaics. Nat Mater.

[CR18] Noone KM, Strein E, Anderson NC, Wu PT, Jenekhe SA, Ginger (2010). Broadband absorbing bulk heterojunction photovoltaics using low-bandgap solution-processed quantum dots. Nano Lett.

[CR19] Peng ZP, Jiang YS, Song YH, Wang C, Zhang HJ (2008). Morphology control of nanoscale PbS particles in a polyol process. Chem Mater.

[CR20] Ridley BA, Nivi B, Jacobson JM (1999). All-inorganic field effect transistors fabricated by printing. Science.

[CR21] Wang SH, Yang SH (2000). Preparation and characterization of oriented PbS crystalline nanorods in polymer films. Langmuir.

[CR22] Wang SF, Gu F, Lü MK (2006). Sonochemical synthesis of hollow PbS nanospheres. Langmuir.

[CR23] Zhou GJ, Lü MK, Xiu ZL, Wang SF, Zhang HP, Zhou YY, Wang SM (2006). Controlled synthesis of high-quality PbS star-shaped dendrites, multipods, truncated nanocubes, and nanocubes and their shape evolution process. J Phys Chem B.

